# Prognostic value of nuclear survivin expression in oesophageal squamous cell carcinoma

**DOI:** 10.1038/sj.bjc.6600696

**Published:** 2003-01-28

**Authors:** P Grabowski, T Kühnel, F Mühr-Wilkenshoff, B Heine, H Stein, M Höpfner, C T Germer, H Scherübl

**Affiliations:** 1 Medical Clinic I, Gastroenterology/Infectious Diseases/Rheumatology, Benjamin Franklin Clinics, FU Berlin, Hindenburgdamm 30, 12200 Berlin, Germany; 2 Institute of Pathology, Benjamin Franklin Clinics, FU Berlin, Hindenburgdamm 30, 12200 Berlin, Germany; 3 Department of Surgery, Benjamin Franklin Clinics, FU Berlin, Hindenburgdamm 30, 12200 Berlin, Germany

**Keywords:** apoptosis, survivin expression, prognostic marker, oesophageal squamous cell cancer, nuclear localisation

## Abstract

Survivin, a new member of the family of apoptosis inhibitors, is expressed almost exclusively in proliferating cells, above all in cancers. Subcellular localisation and prognostic implications of the survivin protein have not yet been determined in oesophageal squamous cell carcinoma. The survival of 84 patients with oesophageal squamous cell carcinomas was correlated with the extent of immunohistochemical survivin expression in tumour cell nuclei. Tumours were scored positive when >5% cells stained positive. Patients were followed up for at least 5 years or until death. In normal oesophageal squamous cell epithelium, some cytoplasmic survivin expression was detected in the basal cells, whereas proliferating cells showed nuclear staining of survivin. Nuclear expression of survivin was also detected in 67 cancers (80%). The mean survival for patients of this group (28 months, range 20–36) was significantly less than that for patients without survivin expression in the tumour cell nuclei (108 months, range 62–154, *P*=0.003). Using univariate analysis, nuclear survivin expression (*P*=0.003), tumour depth (*P*=0.001), lymph node metastasis (*P*=0.003) and stage (*P*<0.001) were the best predictors of survival. In contrast, cytoplasmic survivin staining was noted in 53 (63%) tumours and had no prognostic relevance. In conclusion, the analysis of nuclear survivin expression identifies subgroups in oesophageal squamous cell cancer with favourable (survivin^−^) or with poor prognosis (survivin^+^). We suggest that the determination of nuclear survivin expression could be used to individualise therapeutic strategies in oesophageal squamous cell cancer in the future.

Apoptosis plays an important role in organ homeostasis, eliminating senescent or damaged cells (Vaux and Korsmeyer, 1999). Impairment of apoptosis facilitates the accumulation of gene mutations by prolonging the cell cycle span and promoting resistance to immune-based cytotoxicity (Yang and Korsmeyer, 1996), finally contributing to carcinogenesis (Thompson, 1995).

It has been shown recently that the inhibitor of apoptosis proteins (IAP) are crucial regulators of the molecular mechanisms of apoptosis (Deveraux and Reed, 1999). Among the IAP members, survivin is unique in that it was found to be expressed in foetal tissue and in a variety of human cancers, but not in nonproliferating adult tissues (Ambrosini *et al*, 1997). Survivin inhibits apoptosis in cells exposed to diverse apoptotic stimuli by associating with microtubules of the mitotic spindles (Li *et al*, 1998) and inhibiting caspase-3 and caspase-7 activity (Tamm *et al*, 1998). Overexpression of survivin has oncogenic potential because it may overcome the G2/M phase checkpoint to enforce progression of cells through mitosis. However, the biological functions of survivin, other than its apoptotic effect, are not well understood in human cancer. Expression of survivin protein is highly correlated with more aggressive forms of neuroblastoma (Adida *et al*, 1998) and oral and skin squamous cell carcinoma (Lo Muzio *et al*, 2001), and poor survival in patients with neuroblastoma (Adida *et al*, 1998), colorectal cancer (Kawasaki *et al*, 1998), nonsmall-cell lung cancer (Monzo *et al*, 1999), breast cancer (Tanaka *et al*, 2000) and increasing rates of recurrence in bladder cancer (Sherr and Roberts, 1995). By investigating mRNA expression of survivin, Kato *et al*, (2001) found a significant correlation with a poorer prognosis and a worse response to chemotherapy in oesophageal cancer. Nevertheless, the expression and function of the survivin protein in oesophageal squamous cell carcinoma (SCC) is yet to be determined.

Therefore, we investigated the protein expression of survivin immunohistochemically and determined its prognostic relevance.

## MATERIALS AND METHODS

### Patients

All oesophageal SCC patients, who were oesophagectomised at the Benjamin Franklin University Clinics (Berlin) between 1982 and 1993, were analysed. Staging and 5-year follow-up was complete in 84 cases. Patients who died of postoperative complications within 30 days were excluded. The clinicopathological data are summarised in Table 1

**Table 1:** Clinicopathological data of patients and tumours according to nuclear survivin expression

	**Patients**		**Nuclear survivin**		
	**No.**	**%**	**Positive**	**%**	**P-value**
*Total*	84		67	79.7	
					
Male	60	71.4	50	74.6	0.198
Female	24	28.6	17	25.4	
					
*Age (years)*					
Median	56.8		56.5		0.557
Range	37–84				
					
*T-stage*	84		67		
T1+T2	34	40.5	24	35.8	0.134
T3+T4	50	59.5	43	64.2	
					
*Lymph node metastasis*	83		67		
Node negative	32	38.6	20	29.9	0.001
Node positive	51	61.4	47	70.1	
					
*Grading*	79		63		
G1+G2	35	44.3	29	46	0.54
emsp;G3	44	55.7	34	54	
					
UICC-stage	84		67		
I	8	9.5	4	6	0.006
II	23	27.4	15	22.4	
III	37	44	32	47.8	
IV	16	19	16	23.9	

.

### Immunohistochemistry

Immunohistochemical staining was performed using the standard avidin–biotin–peroxidase kit (DAKO Hamburg, Germany). Tumour sections (2–3 *μ*m), of adjacent normal squamous cell epithelium and of any adjacent high-grade dysplasias, which were found in 10 of the 84 cases, were evaluated. High-grade dysplasias are now recommended by the WHO to be referred to as high-grade intraepithelial neoplasia (Gabbert *et al*, 2000). Briefly, deparaffinised sections were heated for 5 min in 1 mM EDTA buffer in a pressure cooker to reactivate the antigen and were treated 30 min with 3% H_2_O_2_ in methanol to abolish endogenous peroxidase activity. Sections were incubated with 0.25 *μ*g/ml anti survivin antibody (NOVUS Biologicals Inc., Littleton, CO, USA) for 30 min at room temperature. Biotinylated anti-rabbit immunoglobulin followed by avidin–biotin–peroxidase complex were applied. Sections were developed with 2′,4′-diaminobenzidine and haematoxylin counterstaining. For ki-67 immunostaining, monoclonal MIB-1-antibody (DAKO Hamburg, Germany) was used at a 1:1000 dilution and sections were stained by standard immunohistochemical techniques (APAAP method), as described elsewhere (Grabowski *et al*, 2001). Analysis of slides was performed in a blinded fashion by the authors Kühnel, Grabowski and Heine, without knowledge of clinicopathological data. Cases were scored positive when more than 5% of the cells reacted with the anti survivin antibody, as proposed by other authors (Kawasaki *et al*, 1998). The nuclear ki-67 labelling index was expressed as the percentage of positively stained cells with respect to a hundred cells in 10 high-power fields.

### Statistical analysis

The *χ*^2^ test was used for comparison of data between groups. Overall survivals were assessed by the Kaplan–Meier method. The significance of differences in overall survival was calculated by the Mantel–Cox log-rank test. Most biological and pathological variables were used as dichotomised (categorial) variables: tissue infiltration (T1–T2 *vs* T3–T4), lymph node involvement (N0 *vs* N1–N2), grading (G1–G2 *vs* G3) and age (>57 years *vs* <57 years). Univariate analysis was performed with the Cox regression model. Differences were considered to be significant for *P*<0.05. All statistical analyses were performed using SPSS software.

## RESULTS

### Survivin expression in normal squamous cell epithelium of the oesophagus

**Figure 1:**
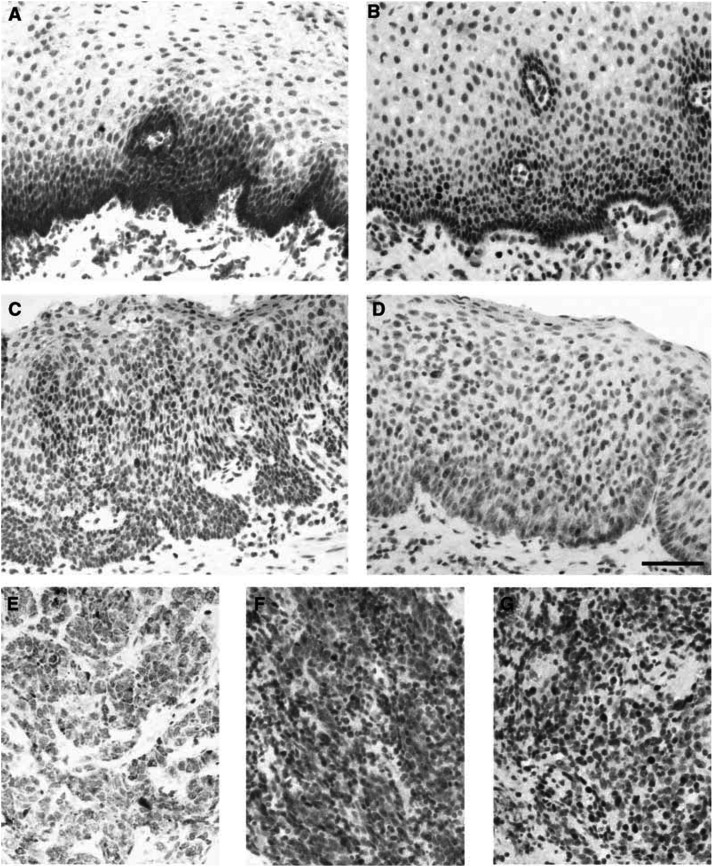
Immunohistochemistry for detection of survivin in (**A**) normal oesophageal squamous cell epithelium, (**C**) high-grade dysplasia (predominant cytoplasmic localisation), (**D**) high-grade dysplasia (predominant nuclear localisation), (**E**) oesophageal SCC (cytoplasmic localisation), (**F**) oesophageal SCC (nuclear localisation). Immunohistochemistry for detection of ki-67 antigen in (**B**) normal oesophageal squamous cell epithelium (paired section to (**A**)) and (**G**) oesophageal SCC (paired section to (**F**)). Scale bar=100 *μ*m.

Survivin expression was detected in all cases of normal squamous cell epithelium of the oesophagus in the basal layer. At the cellular level, survivin staining was predominantly cytoplasmic. Some minimal nuclear immunoreactivity was also observed (Figure 1A

). The staining pattern and intensity of the normal squamous cell epithelium were consistent in different specimens.

### Survivin expression in dysplasia

Survivin was detected in dysplastic epithelial cells (Figure 1C). In contrast to the distribution seen in normal squamous epithelial cells, survivin staining in dysplasia was more intense and uniformly distributed throughout the cytoplasm of all dysplastic cells. Furthermore, the intensity of survivin staining did not show a gradient from the lower layers toward the surface, but was uniform throughout all levels of the lesion. In addition, nuclear expression of survivin was found in some dysplastic cells (Figure 1D).

### Survivin expression in oesophageal SCC

Survivin staining was heterogeneous in oesophageal SCC. In 53 tumours (63%), a cytoplasmic expression of survivin was observed (Figure 1E). A total of 67 tumours (80%) showed a positive nuclear staining for survivin (Figure 1F). Both cytoplasmic and nuclear staining was seen in 46 (55%) cases. Only 10 (12%) tumours did not show any survivin expression at all. The staining within a tumour was often heterogeneous. In areas adjacent to the normal squamous cell epithelium, for example, cytoplasmic survivin expression was present. In more infiltrative parts of the same tumour, nuclear survivin expression occurred.

### Evaluation of cell proliferation by ki-67 immunohistochemistry

All cases were evaluated for the immunohistochemical localisation of ki-67, a marker of cell proliferation. The distribution of ki-67 staining in normal squamous cell epithelium, dysplasia and carcinoma was similar to the distribution of survivin staining. At the individual cellular level, as evaluated in paired serial sections, there was some correlation between survivin and ki-67 staining. However, many more cells were positive for the ki-67 antigen than for survivin (Figures 1B and G).

### Nuclear survivin expression and clinicopathological parameters

Statistical correlation between nuclear survivin expression and sex, age, tumour site or grading revealed no significant differences between survivin^+^ and survivin^−^ oesophageal SCC cases (*χ*^2^ test). However, nuclear survivin expression was significantly increased in primary SCC, which had metastasised into lymph nodes (*P*=0.001) or distant organs (*P*=0.02). Moreover, tumours with nuclear survivin expression presented with higher T-stages, but this trend did not reach statistical significance (*P*=0.08, Table 1).

### Prognostic implications of nuclear survivin expression

**Figure 2:**
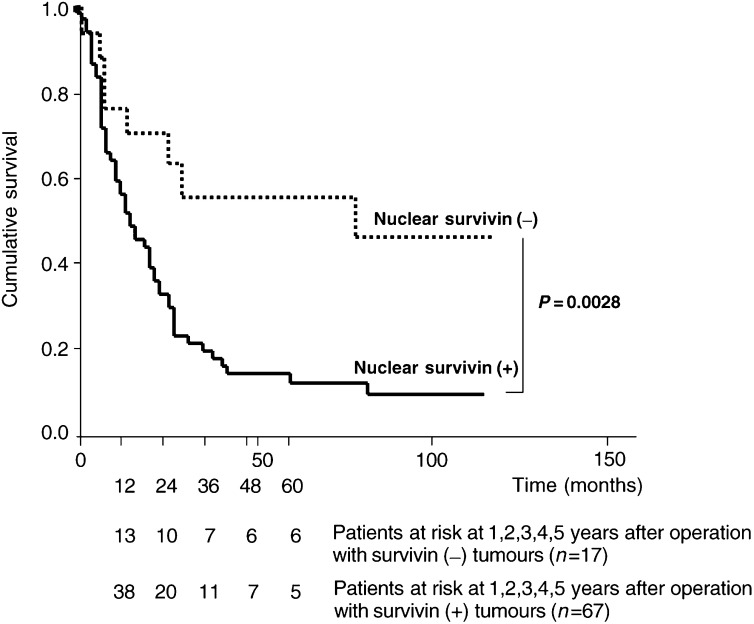
Kaplan–Meier survival curves for positive (*n*=67) and negative (*n*=17) cases of oesophageal SCC regarding nuclear survivin expression (Mantel–Cox log-rank test: *P*=0.0028). Number of patients at risk at 1–5 years after the operation are indicated below the survival curves.

Kaplan–Meier survival curves for patients with oesophageal SCC, categorized according to nuclear survivin expression, are shown in Figure 2

. The mean survival of patients with nuclear survivin expression (28 months, range 20–36) was significantly less than that of patients with no nuclear survivin expression (108 months, range 62–154; *P*=0.0028, Mantel–Cox log-rank test). Using univariate Cox regression analysis, nuclear survivin expression (*P*=0.003), depth of tumour invasion (*P*=0.001), lymph node metastasis (*P*=0.003) and stage (*P*<0.001) were the best predictors of survival of oesophageal SCC patients. In contrast, cytoplasmic expression of survivin had no prognostic relevance.

## DISCUSSION

Survivin, a recently identified member of the family of apoptosis inhibitors, is a bifunctional protein that suppresses apoptosis and regulates cell division (Altieri *et al*, 1999). This protein has attracted great interest as a potential drug target because its expression appears to be tumour-specific (Velculescu *et al*, 1999). While foetal tissues contain abundant survivin mRNA and protein, most nonproliferating adult tissues do not. In contrast, the vast majority of cancers express survivin protein, suggesting that reactivation of survivin gene expression occurs commonly in cancers (Ambrosini *et al*, 1997). Correspondingly, previous reports have shown that the presence of survivin is associated with poor survival among patients with colorectal cancer (Kawasaki *et al*, 1998), non small-cell lung cancer (Monzo *et al*, 1999), breast cancer (Tanaka *et al*, 2000) and neuroblastoma (Adida *et al*, 1998). In our study on 84 patients with oesophageal SCC, we now show that nuclear survivin protein is expressed in 80% of these tumours, thereby confirming and extending earlier results on mRNA expression of survivin in 51 oesophageal SCC patients (Kato *et al*, 2001). Furthermore, we could differentiate between cytoplasmic and nuclear localisation of survivin protein expression and were the first to show a translocation of survivin during carcinogenesis. In normal squamous cell epithelium of the oesophagus, survivin was mainly localised in the cytoplasm of the basal layers. Cytoplasmic staining was also found in some high-grade dysplasia and/or carcinoma, albeit throughout all levels of the neoplasia. Additionally, nuclear survivin expression was observed in some squamous epithelial cells, but much more in high-grade dysplasia and in the corresponding tumours. The restriction of survivin expression to the basal layers of the normal oesophageal squamous cell epithelium suggests that survivin may be related to cell proliferation. Accordingly, we performed serial microsection immunostaining for survivin and ki-67, a marker of cell proliferation, and observed some cellular congruence, but, interestingly, many more cells stained for ki-67 than for survivin. Similar findings were reported in normal colonic mucosa (Gianani *et al*, 2001). One possible explanation is that ki-67 antigen is expressed in every phase of the cell cycle (Endl and Gerdes, 2000), whereas survivin is only present in the G2/M-phase of the cell cycle (Li *et al*, 1998). In this context, the functional role of nuclear survivin has been addressed in hepatocellular cancer (Ito *et al*, 2000). Here, the predominant function of survivin was not the (cytoplasmic) caspase-3-dependent antiapoptotic effect, but the cell cycle phase redistribution. The authors found that survivin promoted cell proliferation by interacting with CDK4 and releasing p21 from CDK4. In line with these findings, it was shown recently (Rodriguez *et al*, 2002) that differences in the amino-acid sequence of the carboxy-terminal domain of survivin determine the different localisation of survivin in the cytoplasm and its splicing variant survivin ΔEx3 in the nucleus. These novel findings suggest that localisation of survivin may constitute an important regulatory mechanism for its role in carcinogenesis and tumour progression.

Accordingly, we show in our study that nuclear survivin expression was nearly invariably associated with metastatic disease to lymph nodes or distant organs. The advanced stage of disease in this subgroup of patients may reflect the rapid growth and aggressiveness of the tumour itself rather than a late diagnosis of a slowly growing tumour. In contrast, cytoplasmic staining of survivin had no prognostic relevance at all. Obviously, the former described antiapoptotic function of survivin is not the predominant effect for tumour progressiveness in oesophageal SCC. Similarly, no prognostic significance of cytoplasmic survivin was found in gastric cancer (Okada *et al*, 2001). Altogether, our data on 84 patients with oesophageal SCC suggest that nuclear survivin expression heralds a particularly poor prognosis. On the other hand, lack of nuclear survivin expression identifies oesophageal SCC patients with a favourable prognosis. Furthermore, we provide evidence that localisation of survivin expression appears to be crucial for its function in tumour cells and again stresses dysregulation of cell cycle as a critical pathogenetic factor in tumour progression.
